# Physical performance and protein-energy wasting in patients treated with nocturnal haemodialysis compared to conventional haemodialysis: protocol of the DiapriFIT study

**DOI:** 10.1186/s12882-017-0562-1

**Published:** 2017-05-01

**Authors:** Manouk Dam, Floor Neelemaat, Trudeke Struijk-Wielinga, Peter J. Weijs, Brigit C. van Jaarsveld

**Affiliations:** 1 VU University Medical Centre, department of Nutrition and Dietetics, Boelelaan 1117, 1081HV Amsterdam, The Netherlands; 2 VU University Medical Centre, department of Nephrology, Boelelaan 1117, 1081HV Amsterdam, The Netherlands

**Keywords:** Nocturnal haemodialysis, Physical performance, Protein-energy wasting, Fibroblast growth factor-23, Sclerostin

## Abstract

**Background:**

Poor physical performance and protein-energy wasting (PEW) are health issues of major concern in haemodialysis patients. The conventional haemodialysis (CHD) regime, three times per week 3–5 h, is subject of discussion because of high morbidity and mortality rates. When patients switch from CHD to longer dialysis sessions, i.e. nocturnal haemodialysis (NHD), improvement in protein intake and increase in body weight is seen. However, it is unclear whether physical performance and more important aspects of PEW, such as body composition, improve as well. Therefore, the aim of this study is to investigate whether physical performance improves and PEW decreases, when patients switch from CHD to NHD. A second aim is to assess the influence of NHD on the biomarkers fibroblast growth factor-23 and sclerostin which are thought to be associated with malnutrition and mortality in patients on haemodialysis.

**Methods:**

This study is a prospective multicentre cohort study with an inclusion aim of 50 patients: 25 patients in a control group (three times per week, 3–5 h CHD) and 25 patients in a nocturnal group (three times per week, 7–9 h NHD). Primary outcome is change in physical performance, measured by the Short Physical Performance Battery. Additional measurements are a 6-min walk test, handgrip strength, a physical activity questionnaire and physical activity monitoring. The secondary outcome of the study is PEW, which will be evaluated by body weight, dual-energy X-ray absorptiometry, bio-electrical impedance spectroscopy, mid-upper arm muscle circumference, subjective global assessment, visual analogue scale for appetite and dietary records. Laboratory measurements including fibroblast growth factor-23 and sclerostin, and quality of life assessed with the Kidney Disease Quality of Life-Short Form are also studied. In every patient, four repeated measurements will be performed during one year of follow-up.

**Discussion:**

This study will investigate whether physical performance improves and PEW decreases when patients switch from CHD to NHD, compared to a control group who continue treatment with CHD. Strengths of this study are the comparison with a conventional haemodialysis cohort, and the broad variety of objective measurements combined with patient-reported outcomes of physical performance and PEW.

**Trial registration:**

NTR4715, Netherlands Trial Register. Registered 30 July 2014.

## Background

Patients with end-stage renal disease (ESRD) are usually treated with a conventional haemodialysis (CHD) regimen, regularly prescribed as 3–4 times per week, 3–5 h haemodialysis sessions. However, prevalence rates of morbidity and mortality remain high, mainly due to cardiovascular complications [1–3]. Therefore, this routine of treatment is being questioned recently [3, 4].

Previous studies suggest that a twice as long haemodialysis treatment, applied as nocturnal haemodialysis (NHD), home or in-center, improves several clinical outcomes compared to CHD [5–8]. For example, improvements are found regarding hypertension and volume control, left ventricular mass, phosphate clearance, hospitalization rates, need for erythropoiesis stimulating agents and antihypertensive medications and survival [9–13]. Apart from cardiovascular complications, poor physical performance and protein-energy wasting (PEW) are risk factors of major concern in ESRD patients and contribute to this high morbidity and mortality [5–8]. Whether physical performance improves after a patient switches to NHD is unclear. Protein intake and body weight, parameters of PEW [14], seem to improve during NHD [15–17]. However, other aspects of PEW, such as muscle mass and mid-upper arm muscle circumference (MUAMC), are scarcely investigated.

Several biomarkers have been identified as risk factors for mortality in ESRD patients. Some of them are associated with bone remodelling and could be influenced by changes in physical activity and body mass. Fibroblast growth factor-23 (FGF-23) is a phosphaturic hormone produced in the osteocytes and is found to be increased in haemodialysis patients [18, 19]. FGF-23 may be associated with low body mass index (BMI) and malnutrition [20, 21]. The protein sclerostin is an important regulator of mineralization both in bone and vasculature. Sclerostin levels increase during progression of kidney disease and in patients on dialysis; however, it is not known whether this is the result of increased sclerostin production or decreased excretion. Studies reporting on associations between sclerostin levels and survival show contradictory results [22–25]. High levels of sclerostin seem associated with higher bone mass and might also have a relation with BMI and body composition [26]. It is of interest to evaluate possible changes in FGF-23 and sclerostin concentrations between ESRD patients who switch from CHD to NHD compared to ESRD patients who remain treated with CHD.

Here, we describe the general design and methodological issues of this study. This study will examine the effects of NHD compared to CHD on multiple intermediate outcome parameters of physical performance and PEW, and on the biomarkers FGF-23 and sclerostin.

## Methods

### Study design

This study is designed as a multicentre prospective cohort study. The NHD group will consist of patients who switch from CHD (2–4 times a week, 3–5 h) to in-centre NHD (2–4 times a week, 7–9 h). The control group will consist of patients who are treated with CHD and continue their treatment. The study protocol is in accordance with the Declaration of Helsinki and has been approved by the Medical Ethics Committee of VU University Medical Centre (VUmc).

### Study population

#### Recruitment of the study population

Patients in the NHD group will be recruited from Diapriva Dialysis Centre Amsterdam, the Netherlands. If a patient qualifies to switch from CHD to NHD depends primarily on the preference of the patient for NHD. Some medical conditions, such as instable angina pectoris, recent acute coronary syndrome, recent epileptic insult and aggressive behaviour are reasons to exclude a patient from NHD. This decision is made by a patient’s nephrologist. The control group will be recruited from VUmc, Diapriva and Spaarne Hospital Hoofddorp, the Netherlands. Patients will be approached by the investigator to inform them about the study and receive written information. Within two weeks patients will be approached by the executive research-dietician to ask for their willingness to participate. The patients who are not interested in NHD because of personal preferences, will be approached for participation in the control group. Patients who are rejected to participate the nocturnal program because of medical reasons, will not be asked to participate in the control group in order to avoid inclusion bias.

#### Inclusion and exclusion criteria

Inclusion criteria for this study are: (1) age ≥18 years, (2) haemodialysis or hemodiafiltration treatment during ≥3 months, with stable haemodialysis regarding weight and blood pressure and absence of infection, (3) written informed consent and (4) ability to understand the study protocol. Patients will be excluded when suffering from: (1) dementia, (2) instable angina pectoris, (3) myocardial infarction in the last 3 months, (4) severe pulmonary disease, (5) a life expectancy of less than 12 months, (6) haemodialysis treatment incompliance, i.e. non-adherence to dialysis regimen, or (7) when they have a planned renal transplantation within 12 months.

### Outcome parameters

The Short Physical Performance Battery (SPPB) is the primary outcome parameter. Secondary outcome parameters are PEW, FGF-23 and sclerostin. Primary and secondary outcome parameters will be measured with a variety of tests, which are listed below (Table 1). All tests will be performed by the executive research-dietician. Baseline parameters will be assessed in duplicate with a 2- to 8-week interval, and after three, six and twelve months of follow-up. The duplicate baseline measurements are done to rule out a possible “learning effect” of the physical performance tests, in this way improving the objectiveness of the results. The mean of the two baseline measurements will be used for analyses. After the baseline measurements the NHD group will switch from CHD to NHD.

**Table 1:** Assessments of physical performance and protein-energy wasting

Assessments	Baseline 1	Baseline 2	Months 3	Months 6	Months 12
Physical performance					
Short physical performance battery	x	x	x	x	x
6- min walk test (m)	x	x	x	x	x
Handgrip strength (kg)	x	x	x	x	x
Physical activity questionnaire	x	x	x	x	x
Physical activity monitor	x	x	x	x	x
PEW					
Body weight (kg) and body mass index (kg/m^2^)	x	x	x	x	x
Dual-Energy X-ray Absorptiometry	-	x	-	-	x
Bioelectrical impedance spectroscopy	x	x	x	x	x
Mid-upper arm muscle circumference (cm)	x	x	x	x	x
Subjective global assessment (score 1–7)	x	x	x	x	x
Dietary intake	-	x	-	-	x
Visual Analogue Scale appetite (cm)	x	x	x	x	x
Other					
Laboratory measurements (e.g. albumin, C-reactive protein)	x	x	x	x	x
FGF-23 and sclerostin	-	x	-	x	x
Quality of life (KDQOL-SF)	x	x	x	x	x
Sleeping behaviour questionnaire	x	x	x	x	x

### Physical performance

#### Short Physical Performance Battery

The SPPB is a broadly used test of physical performance which is validated in various patient groups [27, 28]. The SPPB assesses lower body function with three objective tests: (1) two gait speed tests using a 4-meter walk, with the fastest value for analyses, (2) five repeated chair stands without use of arms and (3) three balance tests, with side-by-side, semitandem and tandem position for 10 s each. Each test is scored from zero to four, higher scores represent better functional status. Total scores are based on the sum of these three components and ranges from zero to twelve [27, 28].

#### Six-minute walk test

A six-minute walk test (6MWT) will be used to objectively assess submaximal functional exercise capacity. The test will be performed indoors, on a walking course of 30 meters, each round representing 60 meters total. The turnaround points will be marked with tape and cones. After a period of 6 min, the distance in meters that a patient has walked will be noted. Patients choose their own walking speed and exercise intensity and if necessary stop and rest during the test [29].

#### Handgrip strength

Handgrip strength (HGS) in kilograms (kg) will be measured using a hydraulic hand dynamometer (JAMAR® 5030 J1, Sammons Preston Rolyan, Sangamon, Chicago, IL) [30]. HGS will be measured in the non-fistula or non-graft arm. Patients with central venous catheters will be measured on both sides (in case a patient will receive vascular access anytime during participation of the study) and if throughout the study the catheter stays in situ the dominant side will be used. The test is performed in sitting position, with elbow flexed at 90° and with a maximal isometric contraction. The test will be repeated and the highest of the two values will be used for analyses.

#### Physical activity

Physical activity will be assessed using the face-to-face LASA Physical Activity Questionnaire (LAPAQ). LAPAQ contains questions about the frequency and duration of several components of activity: walking, cycling, gardening, sports and household activities during the past two weeks, reported in total minutes per day [31, 32]. The total time spent on activities (in minutes) over the last two weeks will be used for analyses.

#### Physical activity monitor

Patients will be asked to wear a physical activity monitor (PAM, PAM B.V., The Netherlands) for seven days. Patients will be instructed to wear the PAM on the right hip. The PAM measures daily physical activity under free-living conditions. PAM is a small-sized and lightweight accelerometer, with a piezoelectric sensor [33]. The PAM measures acceleration in both horizontal and vertical direction and has the ability to store data continuously. The PAM provides a cumulative score, which is a measure for 24 h physical activity (i.e. the PAM score).

### Protein-energy wasting

PEW is determined by criteria which are introduced by the International Society of Renal Nutrition and Metabolism (ISRNM) in 2008 [14]. The following criteria are recommended in four domains: (1) low serum albumin, prealbumin or cholesterol, (2) low body weight, weight loss or reduced body fat, (3) reduced muscle mass, and (4) low energy or protein intake [14]. In this study, we will assess the presence of PEW for every patient according to this definition, using the presence of at least three of the following four criteria as indication of PEW: (1) serum albumin <3.8 g/100 ml, (2) BMI <23 kg/m^2^, (3) reduced MUAMC (>10% compared with the 50^th^ percentile of the reference population) and (4) protein intake <0.8 g/kg/day. We avoided time-dependent criteria for PEW in this assessment, because we are scoring the presence of PEW at short time intervals, during more than one year of follow-up. If we would include dynamic criteria, such as “weight loss over 6 months”, this would entangle the results of measurements at different moments in time and possibly obscure the effect of our treatment modalities. Apart from scoring the presence of PEW, we also will evaluate all different components of the ISRNM PEW-definition separately, in order to establish the value of the combined as well as of the individual parameters.

#### Body weight, height and body mass index

Dry body weight in kg will be used to establish weight. The patient’s nephrologist establishes the dry body weight. In case a patient does not have a dry body weight, measured weight after dialysis treatment will be noted.

Height in centimetres (cm) will be measured using knee height in patients of 55 years and older. For patients younger than 55 years height will be measured with a stadiometer. These two ways to establish height are chosen for two reasons: at first, the aspect of age-related shrinking is relevant in older patients; secondly, for calculating the height by knee height, population-specific formulas are applicable, which are only available for patients from the age of 55 years and older [34]. Knee height will be measured with a knee height calipar [Seca 207, Birmingham, United Kingdom]. For calculating BMI, current dry body weight in kg is divided by the square of height in meters.

#### Dual-energy X-ray absorptiometry

Dual-energy X-ray Absorptiometry (DXA, Hologic Discovery A (S/N84993) with software version APEX 4.5.3.) will be performed to measure total body composition. DXA will be assessed at baseline and after 12 months of follow-up. DXA provides information regarding fat mass in kg (FM), lean tissue mass in kg (LTM) and appendicular skeletal muscle mass in kg (sum of lean mass in arms and legs without bone and fat mass) [35].

#### Fat free mass and fat mass by multi-frequency bioelectrical impedance spectroscopy

Body composition will be measured with bioelectrical impedance spectroscopy (BIS), using a Body Composition Monitor (Fresenius Medical Care, Bad Homburg, Germany). Measurements will be performed at least 30 min after a patient’s haemodialysis treatment, on a mid-week dialysis day, to create optimal stability in a patient’s body fluid compartments. Shoes and socks will be removed and patients will be in supine position. Two electrodes (Fresenius Medical Care, M351431) will be placed on the dorsal surfaces of the hand and two on the foot, both on the side contralateral to the vascular access. Patients with a pacemaker will be excluded from this measurement, due to the possibility of evoking arrhythmias. Measurements will be carried out at 50 frequencies (5–1000 kHz) [36].

#### Mid-upper arm (muscle) circumference

Mid-upper arm circumference (MUAC) indicates a patient’s muscle mass and fat mass. Measurements will be performed using plastic tape, at midpoint of the arm without the vascular access. The mid-point of the arm is halfway between the tip of the shoulder and the tip of the elbow. Next, triceps skinfold (TSF) will be measured using a skinfold caliper (Harpenden, Baty International, West Sussex, United Kingdom) and repeated twice. The mean value of these two measurements will be used for data analyses. Mid-upper arm muscle circumference (MUAMC) is calculated by the equation: MUAMC (cm) = MUAC (cm) − (3.14 × TSF (cm)). These values will be compared to reference values by Frisancho [37].

#### Subjective global assessment

The 7-point Subjective Global Assessment (SGA) is a validated, frequently used tool and includes several components; (1) history of weight loss over the previous six months, (2) dietary intake and gastro-intestinal symptoms (appetite, vomiting, nausea and diarrhoea) and a physical examination of (3) muscle wasting and (4) loss of subcutaneous fat mass. Each of these components are scored by the executive research-dietician using a structured scoring form. In each subscale a patient will at first be classified into one of three categories (severe malnutrition, moderate malnutrition or normal nutritional status). Secondly, the score will be fine-tuned based on several aspects, e.g. how the situation was in the last two weeks. At the end a total score between 1 to 7 points will be noted. A score of 1–2 indicates severe malnutrition, 3–5 moderate malnutrition and 6–7 normal nutritional status [38, 39].

#### Dietary intake

At baseline and after 12 months of follow-up, patients will be asked to keep a 3-day dietary record on a dialysis day, a non-dialysis day and a weekend day. Patients receive instructions from the executive research-dietician how to complete their dietary record and afterwards the dietary record will be analysed by the same research-dietician. From these 3-day records a mean value will be calculated of total energy (kcal and kcal/kg), total protein (grams (g) and g/kg), fat (g), carbohydrate (g), potassium (milligrams (mg)), sodium (mg), phosphate (mg), calcium (mg), dietary fibre (g) and fluids (millilitres (ml)). Sodium intake will be considered as an estimation of true intake, because 24 h urine collection is not reliable in dialysis patients.

#### Appetite score

Visual analogue scale (VAS) for appetite will be used to establish a patient’s appetite. VAS appetite consists of a horizontal 10 centimetre line, each end of the line expressing the most negative (“I had no appetite at all”) and the most positive sensation regarding appetite (“My appetite was very good”). Patients will be asked to express their appetite during the past week by making a mark across the 10 centimetre line [40]. The VAS score is determined by measuring the distance in centimetres from the left end of the line to the point drawn by the patient. A higher score represents a better appetite.

#### Biochemical parameters

Various biochemical parameters will be assessed prior to a midweek dialysis treatment: hemoglobin (mmol/l), urea (mmol/l), creatinine (μmol/l), albumin (g/l), C-reactive Protein (mg/l), calcium (mmol/l), phosphate (mmol/l), iron saturation (%), ferritin (μg/l), parathyroid hormone (PTH, pmol/l), potassium (mmol/l), sodium (mmol/l), normalized protein nitrogen appearance (nPNA, g/kg/day) and total cholesterol (mmol/l). Standard, validated laboratory techniques are used, in order to reflect mean metabolic circumstances. Albumin will be measured before dialysis with either the bromcresol purple or green method. Bromcresol purple values will be converted to bromcresol green values using the following equation: bromcresol green = bromcresol purple + 5.5 (g/L). [41]

#### FGF-23 and sclerostin

FGF-23 levels (RU/ml) will be measured at baseline and after 6 and 12 months follow-up. Ethylenediaminetetraacetic acid (EDTA) plasma samples will be taken, using an aprotinin inhibitor to preserve stability [42]. C-terminal FGF-23 was measured using an enzyme-linked immunosorbent assay (ELISA) kit (Immunotopics, San Clemente, CA, USA). Sclerostin (pmol/l) will also be measured, at baseline and after 6 and 12 months of follow-up, using the TECO sclerostin ELISA kit (TECOmedical AG, Sissach, Switzerland). Blood samples will be collected following the patient’s dialysis treatment and will be processed by the biochemical laboratory within one hour after dialysis treatment. All blood samples will be centrifuged and stored at −80°.

#### Quality of life and sleeping behaviour

Quality of life will be assessed with the validated Kidney Disease Quality of Life-Short Form (KDQOL-SF, Dutch version 1.2). The questionnaire consists of a generic part, based on the Short Form with 36 questions (SF-36), then followed by a disease-specific part. The generic part summarizes scores leading to a physical component summery score (PCS) and a mental component summary score (MCS). The disease-specific part contains different domains regarding effects of kidney disease on daily life, burden of kidney disease, work status, cognitive function, quality of social interaction and sleep [43]. The domains all have a score ranking from 0 to 100, higher scores representing a better quality of life. An additional questionnaire will be given containing 12 questions regarding sleeping behaviour.

### Statistical analyses

#### Statistical power

In advance, we estimated that a clinically significant difference of 10% in physical performance, measured with the SPPB, with a statistical significance level of 0.05 and a power of 80%, and a SD of 1.2-1.5, a sample size of 16–25 patients per group would be required [44]. The power of the study is increased due to four repeated measurements within a patient, which allows for some attrition. Therefore, our aim is including a total of 50 patients, 25 patients per group.

#### Statistical analyses

Patient characteristics will be reported as means with standard deviations, medians with interquartile ranges or proportions when appropriate. For the analyses, the linear mixed regression model will be used. Regression analyses will be adjusted for possible confounders such as gender, age and dialysis vintage. If patients discontinue their participation of the study for any reason, all available data collected until the moment of discontinuation will be included in the analyses. Statistical significance will be defined as *p* ≤ 0.05 and will be performed using the SPSS-system for Windows, version 22.0 (SPSS, Chicago, IL, USA).

## Discussion

This study has several strengths. First of all, it has a prospective design and will include incident NHD patients. The results of NHD will be compared with baseline values, as well as with results of a control group, to account for changes over time and a possible learning effect of certain tests.

Second, physical performance and PEW will be measured using a wide range of objective measurements, combined with patient-reported outcomes. A combination of assessments in both ways will provide the most complete assessment of these outcomes. We expect a broad range of physical conditions in this population, due to e.g. various ages, weights and medical conditions. However, with all of these different tests, patients with marginal physical conditions will not have to be excluded. Also, a strength is the assessment of body composition measured with BIS as well as with a DXA scan which will provide comprehensive information regarding body composition. To our knowledge there are no previous studies investigating physical performance and PEW with this combination of different assessments.

A limitation of this study is a possible negative influence of this extensive battery testing on the willingness to participate to this study: the chronic dialysis population has already the burden of a time consuming dialysis treatment combined with numerous medical appointments. For the NHD patients we expect this to be of minimal influence, because they will be motivated to observe their own progression in physical performance and nutritional status when switching to another dialysis treatment. More difficulties could arise during the recruitment of the control patients, because improvements during the year regarding their physical performance status will probably not be realized. To overcome this barrier, we want to perform as much of the assessments during dialysis, also providing patients some kind of distraction from dialysis. Not all assessments will be feasible to perform during a dialysis treatment, namely the SPPB, BIS and 6MWT. Therefore, these tests will be assessed after a patient has finished the dialysis treatment. This could be another reason for control patients to be reluctant to participate. However, the assessments are easy and quick to perform, so we will emphasize this during recruitment and thereby expect the chance of missing data to be minimized. For the NHD group all tests will be performed after their dialysis treatment, because it will be infeasible to perform these tests during NHD.

Another limitation is the lack of randomization. However, comparing different dialysis modalities is hardly possible by randomization, because patients do not want to participate in such trials. Even large study consortia do not succeed in obtaining large enough patient numbers in NHD trials [13]. To avoid differences in both groups, we will not include patients in the control group that are excluded from NHD due to medical reasons. However, there might be a difference in patients who are motivated to perform NHD in comparison to patients who do not want this treatment, i.e. selection bias. In practice we notice that reasons not to perform NHD are often social reasons, e.g. having kids or a partner and not wanting to be away during the night. However, in order to minimize the effect of bias on the study results, we will correct the results for relevant confounding factors.

In conclusion, this study will examine the effects of NHD compared to CHD on multiple intermediate outcomes of physical performance and PEW and on the biomarkers FGF-23 and sclerostin.

## Abbreviations

6MWT: 6-min walk test
BIS: bioelectrical impedance spectroscopy
BMI: body mass index
CHD: conventional haemodialysis
DXA: dual-energy X-ray absorptiometry
EDTA: ethylenediaminetetraacetic acid
ESRD: end-stage renal disease
FGF-23: fibroblast growth factor-23
FM: fat mass
HGS: handgrip strength
ISRNM: international society of renal nutrition and metabolism
KDQOL-SF: kidney disease quality of life-short form
LAPAQ: LASA physical activity questionnaire
LTM: lean tissue mass
MUAC: mid-upper arm circumference
MUAMC: mid-upper arm muscle circumference
NHD: nocturnal haemodialysis
nPNA: normalized protein nitrogen appearance
PAM: physical activity monitor
PEW: protein-energy wasting
PTH: parathyroid hormone
SGA: subjective global assessment
SPPB: short physical performance battery
VAS: visual analogue scale
